# 3D saturation recovery imaging for free breathing myocardial T1 mapping

**DOI:** 10.1186/1532-429X-15-S1-P44

**Published:** 2013-01-30

**Authors:** Markus Henningsson, Rene M Botnar, Tobias Voigt

**Affiliations:** 1 Division of Imaging Sciences and Biomedical Engineering, KIng's College London, London, UK; 2 Philips Research Europe, Hamburg, Germany

## Background

Longitudinal magnetization relaxation time (T1) mapping can overcome limitations of late gadolinium enhancement for the detection of diffuse fibrosis in the myocardium. Several T1 mapping methods have been proposed in recent years; however most of them are limited to 2D breath-hold acquisitions with associated limitations in signal-to-noise ratio (SNR) and spatial resolution. A MOdified Look-Locker Inversion recovery (MOLLI) approach has been widely used, however systematic T1 errors have been reported which can be overcome by using a Saturation recovery single SHot Acquisition (SASHA). In this work, we extend the 2D SASHA to 3D using a 1D diaphragmatic navigator for respiratory motion correction and segmented k-space acquisitions. The proposed free breathing 3D SASHA method was compared to breath-hold 2D SASHA and 2D MOLLI in healthy volunteers.

## Methods

The 3D SASHA sequence was implemented as a radiofrequency spoiled gradient echo sequence. Nine 3D images were acquired at different delay times (0 to 700 ms) from the saturation pulses, plus an image acquisition prior to any saturation pulses to estimate the magnetization after an infinite delay. Imaging parameters of the 3D SASHA sequence were; FOV=300×300×80 mm^3^, spatial resolution=1.5×1.5×8 mm^3^, α=30°, TR/TE=5.2/2.6 ms, nominal scan time=5:10 min. A 6 mm window and 0.6 tracking factor was used for the respiratory gating and slice tracking. 6 healthy volunteers were scanned on a 1.5T Philips scanner.

## Results

**Figure 1:**
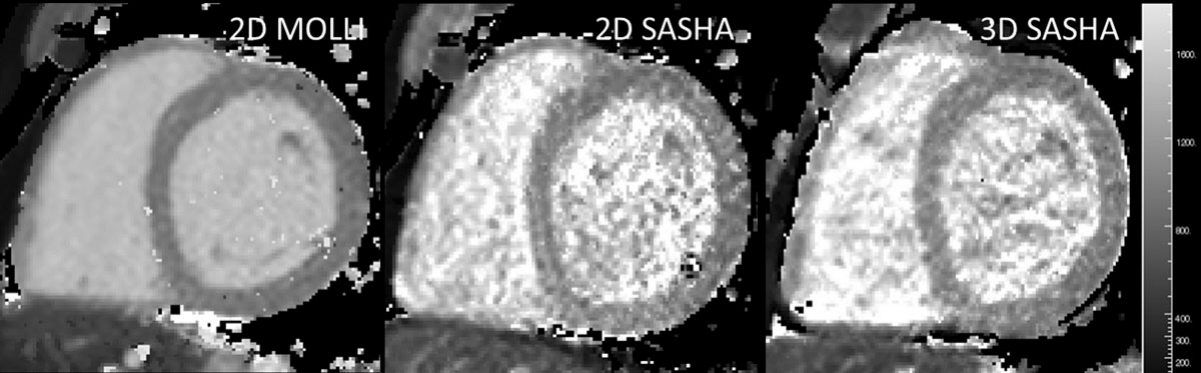
T1 maps from one healthy volunteer. 2D MOLLI and 2D SASHA acquired during breath holding. 3D SASHA acquired during free breathing. All T1 maps are windowed to the same dynamic range (0 < T1 < 2000).

**Figure 2:**
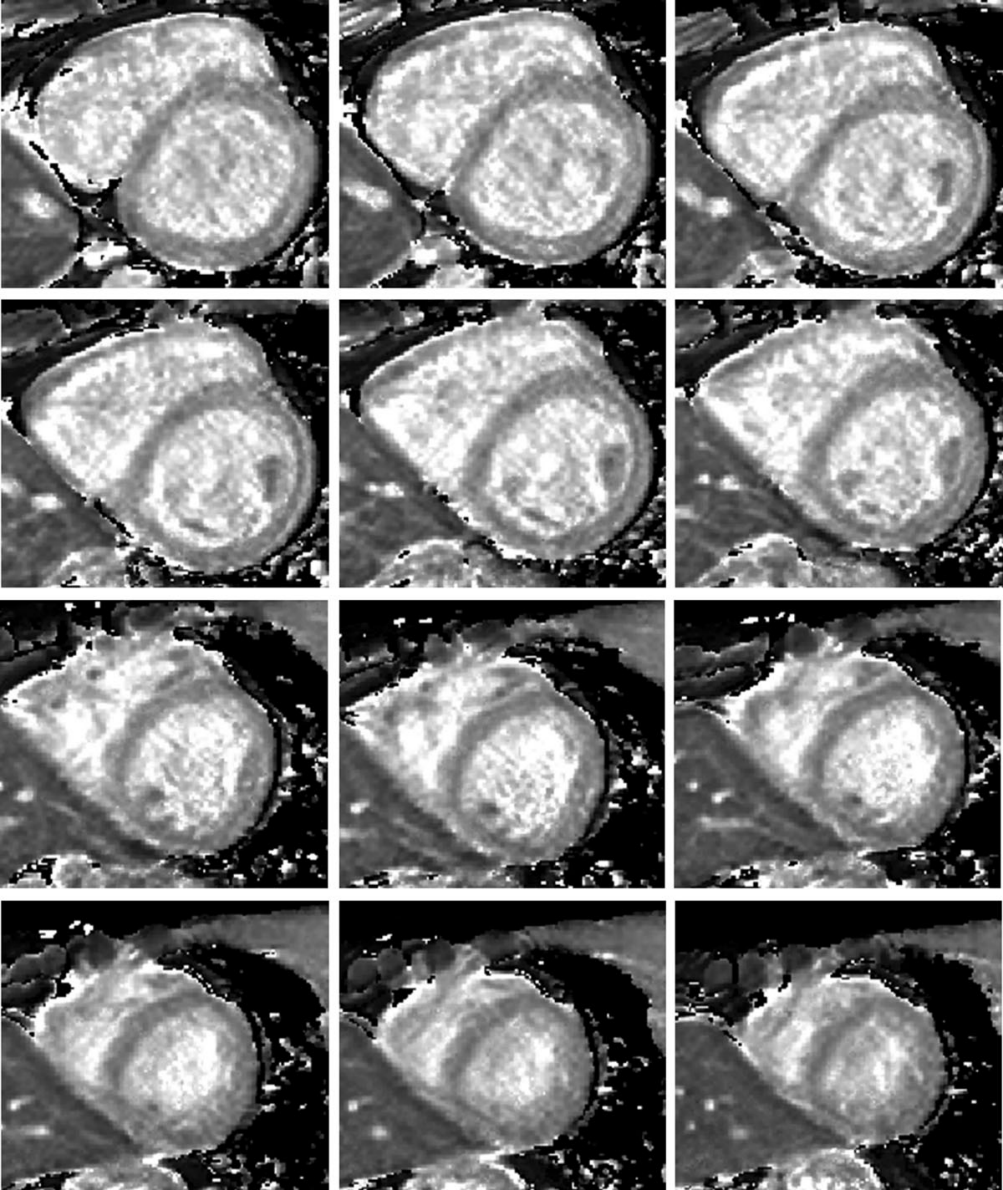
Whole left ventricle T1 map, from base to apex, acquired during free breathing using 3D SASHA.

Figure 1 shows short-axis T1 maps using 2D MOLLI, 2D SASHA and 3D MOLLI from a healthy volunteer. The average myocardial T1 values for the 6 healthy subjects were: 2D MOLLI=944±14 ms, 2D SASHA=1105±46 ms and 3D SASHA=1060±20 ms. Average T1 values for blood were: 2D MOLLI=1501±40 ms, 2D SASHA=1621±168 ms and 3D SASHA=1640±120 ms. T1 values were larger for the SASHA acquisitions than for MOLLI, which is due to the underestimation of T1 of the MOLLI sequence; however the agreement between 2D and 3D SASHA was excellent. Due to the 3D encoding, the SNR of 3D SASHA was higher than 2D SASHA (Figure 1) in an otherwise SNR-starved sequence, as saturation pulses and relatively short delay times are used in the SASHA sequence. Figure 2 shows a 3D SASHA T1 map of the whole left ventricle, acquired in 10:15 mins. The average scan time of the 3D SASHA sequence for the 6 volunteers was 9:37±0:41 mins.

## Conclusions

3D SASHA allows for accurate T1 quantification of the whole left ventricle in a clinically acceptable scan time. It overcomes the need for breath hold acquisitions and improves the SNR compared to 2D SASHA.

## Funding

British Heart Foundation: RG/12/1/29262

